# Transition-Metal Doped Ceria Microspheres with Nanoporous Structures for CO Oxidation

**DOI:** 10.1038/srep23900

**Published:** 2016-03-31

**Authors:** Lin Zhou, Xiaoxiao Li, Ze Yao, Zhuwen Chen, Mei Hong, Rongshu Zhu, Yongye Liang, Jing Zhao

**Affiliations:** 1 Shenzhen Key Lab of Nano-Micro Material Research, School of Chemical Biology & Biotechnology, Peking University Shenzhen Graduate School, Shenzhen, 518055, China; 2 Department of Materials Science & Engineering, South University of Science & Technology of China, Shenzhen, 518055, China; 3 Environmental Science and Engineering Research Center, School of Civil and Environmental Engineering, Harbin Institute of Technology Shenzhen Graduate School, Shenzhen, 518055, China; 4 State Key Laboratory of Pharmaceutical Biotechnology, Institute of Chemistry and Biomedical Sciences, School of Life Sciences, Nanjing University, Nanjing, 210093, China

## Abstract

Catalytic oxidation of carbon monoxide (CO) is of great importance in many different fields of industry. Until now it still remains challenging to use non-noble metal based catalysts to oxidize CO at low temperature. Herein, we report a new class of nanoporous, uniform, and transition metal-doped cerium (IV) oxide (ceria, CeO_2_) microsphere for CO oxidation catalysis. The porous and uniform microsphere is generated by sacrificed polymer template. Transition-metals, like Cu, Co, Ni, Mn and Fe, were doped into CeO_2_ microspheres. The combination of hierarchical structure and metal doping afford superior catalytic activities of the doped ceria microspheres, which could pave a new way to advanced non-precious metal based catalysts for CO oxidation.

Catalytic oxidation of carbon monoxide is of great importance in a variety of practical applications, such as automobile exhaust purifiers, CO gas masks and CO sensors^1^. McKinney first reported transition metal oxides as catalysts for oxidation of CO^2^. Since then, noble metals, especially Pt, Pd and Au, are widely employed for catalytic oxidation of CO^3^. For example, Pt/Al_2_O_3_, and Pt/A-zeolite were utilized in the catalytic oxidation of CO^4^. Pd-based ceria materials were synthesized and showed excellent activities in the oxidation reaction^5^. Modified Au/CeO_2_was fabricated through a nanocasting route and a high catalytic activity was obtained by the formation of inter-particle bridges^6^. Structure-activity relationships of metal doped nano-Au catalysts were then elucidated in Reddy’s work^7^. CO oxidation mechanisms were studied and metal-support interaction between nanoparticles and the supports partly accounted for the improvement of catalytic activities^8^,^9^,^10^. However, those noble metal based catalysts are still expensive, scarce and sensitive to impurities for deactivation^11^, which hinder their widespread applications.

Thus, low cost alternatives based on nonprecious metals, like Cu, Co, Ni, Zn and Mn, have been studied over decays^12^. A CuO-CoO_x_ hybrid ceria system also showed good performance in CO oxidation reaction^13^. Thermally stable Fe-Cu/CeO_2_ catalysts were prepared for CO oxidation^14^. Recently, CO oxidation at low temperature with nonprecious metal based catalysts was an important research goal^15^,^16^,^17^,^18^. Madras and his co-workers studied reaction dynamics of Sn doped transition-metal (Cu, Fe, Co, Mn) oxides in CO oxidation, which utilized more cheap metals in the low-temperature CO oxidation reaction^19^. Mn-Ce solid solution supported on alumina helped to improve the activity of Mn-based catalysts, showing outstanding catalytic activity at low temperatures^20^. Schüth and his coworkers developed Co doped nanocomposites with unusual catalytic behavior through template based method^21^. Among different types of metal oxide supports, cerium oxides were widely investigated in industrial applications^22^,^23^,^24^,^25^ due to its intrinsic physical and chemical properties^26^,^27^,^28^. Substitution of metal cations into ceria could lead to the formation of structural defects or mixed cerium oxide phases, thus having effects on the activities of oxygen ion^29^. Therefore, doping of cheap transition metals into ceria catalysts is of fundamental importance in low temperature CO oxidation.

The morphology and structure of metal and ceria nanoparticles are important for catalytic performance, but are hard to be controlled^30^,^31^,^32^,^33^,^34^. Surfactants or silica template were usually involved in the synthesis of metal/metal oxide hybrids with well-defined structures. It was noteworthy that catalysts prepared through soft template method without further treatment were not as active as those fabricated through hard template method^35^. Recently we developed a hard template method to synthesize transition-metal doped CeO_2_ microsphere by employing microsized and porous P (GMA-*co*-EGDMA) polymer sphere as template^36^. The size and pore structures of the polymer template can be precisely controlled during the process of swelling polymerization. The surface of the polymer templates can be functionalized with quaternary ammonium groups by ring-opening reaction of epoxide groups, which could introduce interaction sites for functional materials. The porous ZrO_2_, Pt/CeO_2_ microspheres prepared by such templates showed superior catalytic activities in the Friedel-Crafts alkylation of indoles and catalytic reduction of 4-nitrophenol^37^.

Herein, we employed such polymer templates to construct monodisperse and uniform CeO_2_ microspheres, and then further modified the materials by metal doping. Several different metal dopants such as Cu, Co, Ni, Fe and Mn were studied. It was found that Cu doped porous CeO_2_ microspheres exhibited the highest catalytic activity, which was much better than that of the undoped CeO_2_ microspheres or the counterparts with ill-defined structures. This new approach of constructing transition metal hybrid nanoporous metal oxide microspheres showed high potential in the development of CO oxidation catalysts.

## Results and Discussion

### Fabrication and Characterization of Transition-metal Doped CeO_2_Microspheres

**Figure 1:**
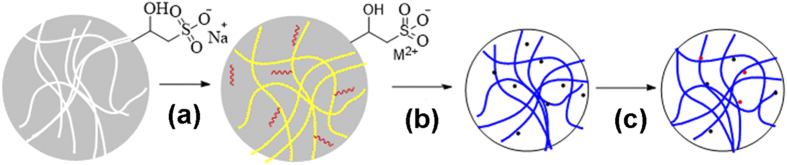
Synthetic scheme of transition metal doped CeO_2_ microspheres: (**a**) sol-gel process; (**b**) calcination; (**c**) H_2_ activation.

**Figure 2:**
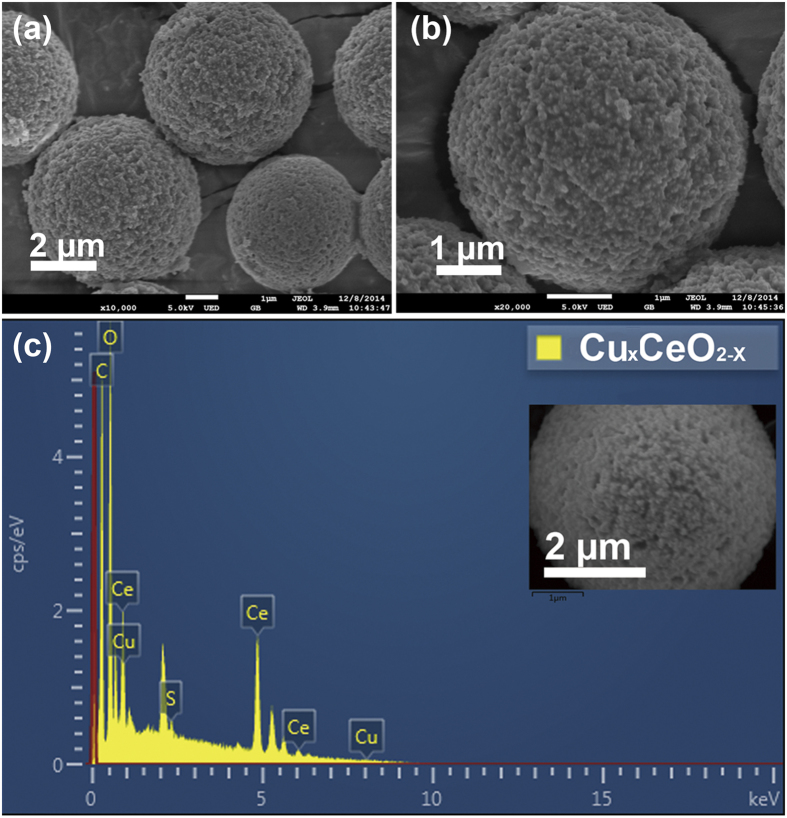
SEM images (**a,b**) and EDS-mapping (**c**) of Cu_x_CeO_2−X_ microspheres.

The synthetic scheme of the transition-metal doped CeO_2_ hybrid microspheres was shown in Fig. 1. To render the surface negatively charged, epoxide groups on the surface of the template (PGMA-*co*-EGDMA) microspheres underwent ring-opening reaction with sodium sulphite (Na_2_SO_3_) at 70 °C, obtaining the sulfonated polymer microspheres. During the sol-gel process, the functionalized polymer microspheres, together with metal salts, and cerium (III) nitrate were dispersed in water and thermally treated at 60 °C in the drying oven for at least 6 h. Species and contents of metals could be adjusted in this step by varying the kinds and amounts of the metal precursors. Transition-metals including Cu, Co, Ni, Mn and Fe could all be incorporated into ceria microspheres in the sol-gel process. Hybrid ceria materials with metal contents of 10 mol% were denoted as M_x_CeO_2−X_ (M_x_ stands for the doping metal). Metal oxide nanoparticles developed and cerium (IV) oxide microspheres formed after calcination in a muffle furnace at 600 °C, during which process polymer template was removed. After H_2_ activation at 330 °C, activated metal hybrid ceria microspheres were successfully synthesized, which was ready for the following heterogeneous catalysis. The scanning electron microscopy (SEM) images of metal doped ceria microspheres were shown in Fig. 2a,b and Supplementary Fig. S1, indicating microsized and monodisperse morphologies of the resulting Cu_x_CeO_2−X_ microsphere and other ceria hybrids, which were similar to those of the parent microspheres and CeO_2_ microspheres without metal-doping. There were no obvious ceria NPs outside the transition metal substituted ceria microspheres after calcination according to Fig. 2b, since cerium and Cu precursor gels were adsorbed into the polymer microspheres during the sol-gel process^36^. EDS-mapping analysis of an individual Cu_x_CeO_2−X_ microsphere was shown in Fig. 2c, indicating the existence of Cu element. The estimated content of Cu in the Cu_x_CeO_2−X_ microspheres was 10 mol% according to element content analysis by ICP-OES.

**Figure 3:**
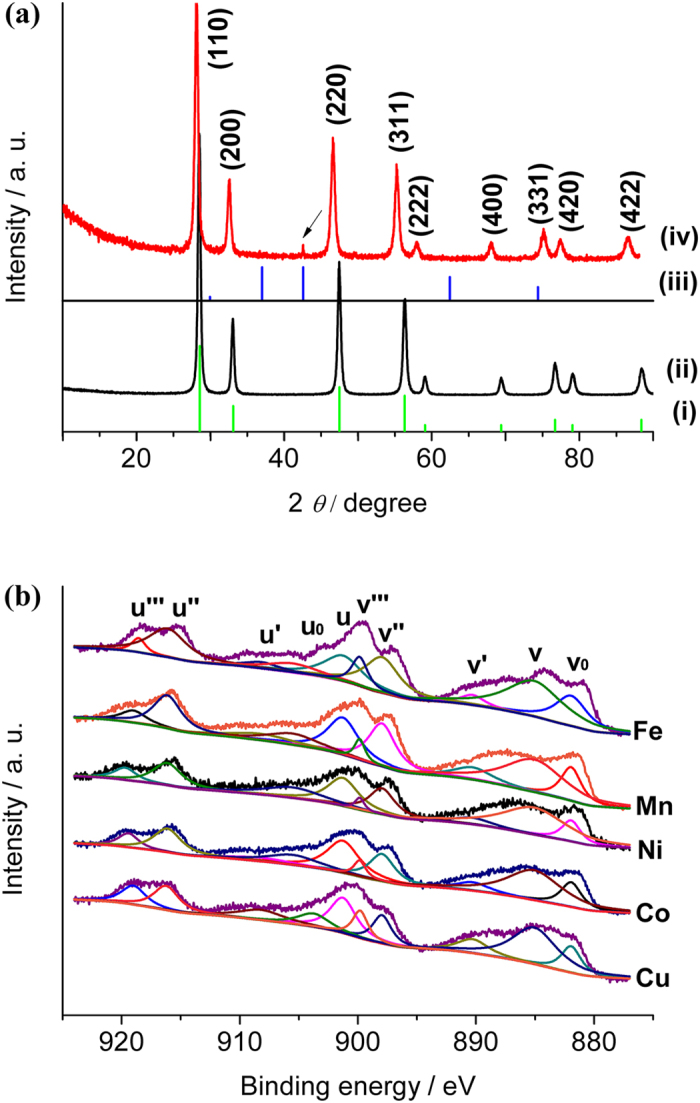
(**a**) XRD patterns of: (i) CeO_2_ standard card (JCPDS No. 34–0394), (ii) CeO_2_ microspheres, (iii) Cu_2_O standard card (JCPDS No. 34–1354) and Cu_x_CeO_2−X_ microspheres (The black arrow indicating diffraction peak from substituted Cu_2_O.) and (**b**) XPS of M_x_CeO_2−X_ microspheres at the Ce 3*d* region: Cu_x_CeO_2−X_, Co_x_CeO_2−X_, Ni_x_CeO_2−X_, Mn_x_CeO_2−X_ and Fe_x_CeO_2−X_.

Particle size distribution analysis of CeO_2_ microsphere and transition metal doped microspheres, like Cu_x_CeO_2−X_, Co_x_CeO_2−X_, Mn_x_CeO_2−X_, Fe_x_CeO_2−X_ and Ni_x_CeO_2−X_ microspheres, was illustrated in Supplementary Fig. S2. The sizes of all the metal oxide microsphere were in the range from 3 to 5 μm, smaller than that of the polymer templates. This could be possibly due to the higher densities of CeO_2_ microspheres than that of polymer microspheres. Pore structure (BET surface area, BET average pore size, *t*-plot micropore area, BJH pore volume) of the metal substituted CeO_2_ hybrids was quantitatively evaluated by N_2_ adsorption/desorption isotherm, which was shown in Supplementary Table S1. BET surface areas of these five kinds of metal substituted ceria microspheres were between 10–20 m^2^/g, which was similar to that of ceria microspheres. Metal contents of the transition-metal substituted ceria hybrids were evaluated through atomic absorption spectroscopy with an optical emission spectrometer (ICP-OES) and scanning electron microscopy energy-dispersive X-ray spectroscopy (SEM-EDS) mapping (Supplementary Table S1) respectively and values calculated from ICP-OES were used to estimate the amounts of metal dopants in the ceria hybrids. The average transition metal substitutions of the Cu_x_CeO_2−X_, Ni_x_CeO_2−X_, Mn_x_CeO_2−X_ and Co_x_CeO_2−X_ hybrids were 10 mol%. FT-IR spectra of Cu_x_CeO_2−X_ microspheres before and after H_2_ reduction were shown in Supplementary Fig. S3, which were similar to that of CeO_2_ with peak at 1126 cm^−1^ 26. The XRD patterns of all the as-synthesized transition metal substituted CeO_2_ microspheres were shown in Fig. 3a. The characteristic diffraction peaks of CeO_2_ microspheres with the face-centered cubic structure indicated good crystallinity (JCPDS No. 34–0394). After Cu substitution, diffraction peaks of the hybrid CeO_2_ materials appeared to shift to lower degrees due to the replacement of Ce^4+^ by larger sized Ce^3+^ 38. A small diffraction peak of Cu_2_O (200) was also observed (JCPDS No. 34–1354), possibly due to the partial reduction of Cu spieces in the Cu_x_CeO_2−X_ hybrid microspheres after thermal treatment under H_2_/N_2_ atmosphere. XPS analysis of Cu 2*p* region in Supplementary Fig. S4 revealed more information about the valence state of Cu. There existed a peak position at 932.1 eV, which could be assigned to Cu 2*p* 3/2, demonstrating the existence of Cu (I) species. Peak located at 935.6 eV could be corresponded to Cu (II) 2*p* 3/2. A strong shakeup at 943.8 eV further confirmed the existence of Cu^2+^ in the Cu_x_CeO_2−X_ hybrid microspheres. Therefore, both Cu (II) and Cu (I) existed in our Cu_x_CeO_2−X_ microspheres, but there was no obvious phase separation for a Cu_x_CeO_2−X_ microsphere (Supplementary Fig. S5). The shift of XRD peaks was also observed in different metal doped ceria samples, suggesting the successful incorporation of the metals into the ceria lattice (Supplementary Fig. S6). XPS at the ceria 3*d* region of metal substituted microspheres was shown in Fig. 3b. By means of XPS-peak-differentiation-imitating analysis, ten different peaks (v_0_, v, v′, v″, v′′′, u_0_, u, u′, u″, u′′′) were obtained, which were corresponded to Ce^3+^ and Ce^4+^ respectively^39^. Oxygen vacancy content (x) was estimated to be half of the concentration of Ce^3+^ ions. Formula shown below illustrated the calculation of Ce^3+^ ions and oxygen vacancy content: x (%) = (v_0_ + v′ + u_0_ + u′)/2 (v_0_ + v + v′ + v″ + v′′′ + u_0_ + u + u′ + u″ + u′′′). (1) Here, peak area of these ten peaks was used in the calculation of oxygen vacancies. Different oxygen vacancies of those transition metal substituted microspheres were listed in Supplementary Table S2. According to the calculated oxygen vacancies, chemical formula of those doped CeO_2_ microspheres were Cu_0.15_CeO_1.85_, Co_0.11_CeO_1.89_, Ni_0.13_CeO_1.87_, Mn_0.14_CeO_1.86_ and Fe_0.12_CeO_1.88_. Among them, oxygen vacancy of Cu doped hybrid microsphere was better than the other catalysts. At the same time, oxygen vacancies in the catalysts gave evidence to the fact that transition-metal spieces were doped into the lattice of ceria. Raman spectra of these metal doped ceria microspheres were shown in Supplementary Fig. S7. Band at 464 cm^−1^ was assigned to F_2g_ mode of CeO_2_^40^. Oxygen vacancies were obviously observed in the range of ~580–600 cm^−1^ 41. We observed that the band at 464 cm^−1^ shifted to lower wavenumbers after metal doping, which could be explained by lattice expansion. Formation of Ce^3+^ in the CeO_2_ lattice was one of the factors, which corresponded with the reason for peak shifts in XRD. Oxygen vacancy was evaluated as half of the concentration of Ce^3+^ ions, which supplemented the analysis of XPS. Heterogeneous metal doping made a big effect on the concentration of Ce^3+^ ion, which could be supported by XRD, XPS and Raman, thus leading to changes in the oxygen vacancies. Cu_x_CeO_2−X_ possessed the largest peak shift at 460 cm^−1^ in the Raman spectrum and the largest oxygen vacancy calculated from XPS, which was the best catalyst in the CO oxidation reaction among all these transition metal doped microspheres. Oxygen active species were more stable on the surface of ceria based catalysts than the bulk and helped to promote the CO oxidation^42^.

### Catalytic Study of Transition-metal Doped CeO_2_ Microspheres

**Figure 4:**
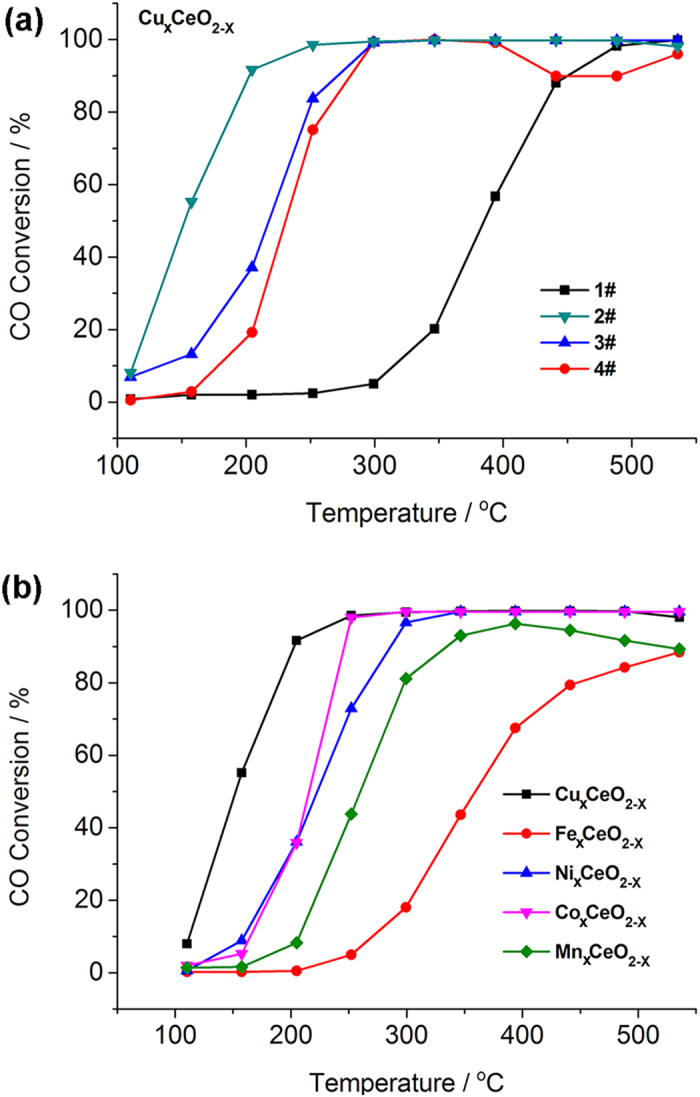
CO catalytic oxidation on metal doped hybrid ceria microspheres. CO conversion curves of (**a**) 1#: CeO_2_ microspheres; 2#: Cu_x_CeO_2−X_ microspheres (10 mol%); 3#: Cu_x_CeO_2−X_ composite (10 mol%); 4# Cu_x_CeO_2−X_ microsphere without H_2_ activation (10 mol%); and (**b**) different metal doped ceria catalysts: Cu_x_CeO_2−X_ (10 mol%), Fe_x_CeO_2−X_ (10 mol%), Ni_x_CeO_2−X_ (10 mol%), Co_x_CeO_2−X_ (10 mol%) and Mn_x_CeO_2−X_ (10 mol%) microspheres.

Catalytic oxidation of CO was performed on Cu doped CeO_2_ microspheres to investigate the influence of prepared procedures on the catalytic performance. CO conversion curves of those Cu_x_CeO_2−X_ hybrids under oxygen rich conditions (2400 ppm CO, 15 vol% O_2_) were shown in Fig. 4a. Introduction of Cu into the CeO_2_ lattice effectively promoted CO catalytic activities compared with CeO_2_ microspheres. A Cu_x_CeO_2−X_ composite with ill-defined structure was synthesized through similar approach to that of Cu_x_CeO_2−X_ microspheres except that no polymer templates were added. SEM images of Cu_x_CeO_2−X_ composite were shown in Supplementary Fig. S8 and no microsphere structure was observed. T_*100*_ represented the temperature at which 100% of CO was converted to CO_2_ and T_*50*_ was the light-off temperature. *T*_50_ of Cu_x_CeO_2−X_ composite was 68 °C higher than that of Cu_x_CeO_2−X_ microspheres, suggesting that the catalytic activity of Cu_x_CeO_2−X_ composite was lower than that of Cu_x_CeO_2−X_ microsphere. It indicated the advantage of the porous microsphere structure in enhancing the catalytic activity.

Without H_2_ activation, *T*_50_ of Cu_x_CeO_2−X_ microspheres was raised to 230 °C, which was 81 °C higher than those with H_2_ activation. At 330 °C under H_2_/N_2_ atmosphere, the Cu species doped in the microspheres were likely to be reduced into lower valence states, such as Cu (I)^36^,^43^, which could help to improve the catalytic activities for CO oxidation^44^. The doping content of Cu on the catalytic performance was also studied (Supplementary Fig. S9). The catalytic activities increased with the increase of Cu content from 2 mol% to 10 mol%, but further increase of Cu content after that led to decrease of the catalytic activity. T_*100*_ and T_*50*_ of all these catalysts mentioned above in Fig. 4 and Supplementary Fig. S9 were listed in Supplementary Table S3. It suggested that Cu dopant was important for the enhancement of CO oxidation catalysis. However, a much larger content (like 44 mol%) of Cu would afford phase separation from the ceria lattice, which might decrease the catalytic performance.

The catalytic stability of Cu_x_CeO_2−X_ was also studied. After cycling catalytic oxidation of CO below 300 °C without activation, the CO conversion was maintained 100% after 4 cycles, and *T*_100_ of the Cu_x_CeO_2−X_ microspheres remained 300 °C after 4 cycles (Supplementary Fig. S10). It revealed the good stability and cyclability of Cu_x_CeO_2−X_ microspheres under the relatively high temperature. The effect of doping metal species on CO oxidation catalysis was further studied. The 100% conversion temperature of the metal doped samples was sequenced as below in Fig. 4b: Cu_x_CeO_2−X_ (300 °C) = Co_x_CeO_2−X_ (300 °C) < Ni_x_CeO_2−X_ (350 °C) < Mn_x_CeO_2−X_ (600 °C) < Fe_x_CeO_2−X_ (above 600 °C). *T*_50_ revealed more detailed activities of the hybrids. *T*_50_ of Cu_x_CeO_2−X_ was 150 °C, which was much lower than the other 4 kinds of metal doped ceria microspheres. Therefore, Cu_x_CeO_2−X_ showed the best catalytic activity of all these hybrid ceria microspheres in the catalytic oxidation of CO. None of these hybrid ceria catalysts underwent obvious sintering or phase changes in the catalysis, suggesting high potential in real-world CO oxidation converters or respirators. It has been reported that Cu-based ceria catalysts show the best CO catalytic activity among cobalt, copper, manganese, nickel, chromium, iron, and vanadium based ceria materials^45^. The superior activity in Cu_x_CeO_2−X_ may be related to the electronic structure^39^ or oxygen vacancies and the detailed mechanism will be addressed in future studies.

## Conclusion

In summary, we have presented a simple and general route for the fabrication of metal nanoparticles deposited on uniform and porous ceria microspheres by employing poly (GMA-*co*-EGDMA) microspheres as hard template. The nanoporous, hierarchical and microsized M_x_CeO_2−X_ hybrid structure exhibited high catalytic activity and good recycling stability, as well as easy recovery. This general synthetic method could furnish uniform and porous metal oxide microspheres embedded with metal nanoparticle, opening door to advanced catalysts in various catalytic applications.

## Experimental Section

### Characterization Techniques

The morphology and structure of the transition-metal substituted CeO_2_ microspheres were observed by field emission scanning electron microscope (FESEM) on a Hitachi S4800 scanning electron microscope (Japan). The particle size distribution analysis was performed with a coulter counter Multisizer 3 (Germany). BET (Brunauer-Emmett-Teller) surface area, t-Plot micropore area, BJH (Barrett-Joyner-Halenda) pore volume, and N_2_ adsorption/desorption of the microspheres were measured on a Micromeritics Tristar II 3020 v1.03 analyzer (USA) at liquid nitrogen temperature (−196 °C). Samples were subjected to vacuum system and then kept at 120 °C for 12 h under vacuum prior to the measurement. FT-IR spectra were collected on a Shimadzu IR Prestige-21 with resolution of 4 cm^−1^. Powder X-ray diffraction (XRD) was recorded by using a Rigaku D/Max-2200PC diffractometer. The diffraction angle range was 2*θ* = 10–80°, with Cu K*α* radiation at 40 KV, 200 mA. X-ray photoelectron spectroscopy (XPS) was measured on a Thermo scientific ESCALAB 250XI (USA) with a monochromatic Al K*α* (1486.6 eV) radiation source. Inductively coupled plasma optical emission spectroscopy (ICP-OES) was operated on ShimadzuICPS-7510 (Japan). Raman was observed on Horiba LabAM HR800 (Japan).

### Procedures for the Preparation of Transition Metal-Doped Hybrid Ceria Microspheres

To a suspension of 1 g of the sulfonated microspheres in 5 mL water, 2 g of Ce(NO_3_)_3_·6H_2_O was added into the mixture. At the same time, metal precursors were added. The mixed suspension was transferred to oven set at 60 °C and then heat-treated for 6 h. Finally, the obtained poly (GMA-*co*-EGDMA)/cerium microspheres with metal cations were calcined at 600 °C for 12 h. After that, metal oxide nanoparticles/CeO_2_ microspheres were obtained. Microspheres were then further treated at 330 °C for 3 h in flowing H_2_/N_2_ (0.5/99.5 v/v) at a heating rate of 10 °C/min.

### Catalytic Study

CO catalytic oxidation measurement was conducted with a fixed-bed continuous flow reactor by temperature programmed reaction (TPR) technique. 0.10 g of the catalysts was carefully held in a 6 mm (i. d.) quartz tubular reactor. A thermocouple was placed in the region of the catalyst bed to monitor the reaction temperature. The temperature was controlled by a PID-regulation system (Bachy, CKW-2200), which was raised at a rate of 4 °C/min from 80 °C to 550 °C. The reaction gas containing CO (2400 ppm) and O_2_ (15 vol%) and balance Ar was fed through the catalyst bed at a rate of 100 mL/min.

The residence time and flow of CO were 0.5 s and 2 cm/s respectively. The compositions of CO, CO_2_ and CO_X_ (=CO + CO_2_) were continuously detected on-line by GC-2014C gas chromatograph (GC) equipped with a column packed with Porapak-Q and a FID detector. The percent conversions were the values calculated according to the equation: % Conversion = 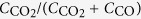 * 100%, where 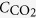 and *C*_CO_ represented the concentrations of CO_2_ and CO, respectively. The concentrations of the feed and the output gases were determined and calculated from the relative peak areas of CO_2_ and CO with respect to the internal Ar standard.

## Additional Information

**How to cite this article**: Zhou, L. *et al.* Transition-Metal Doped Ceria Microspheres with Nanoporous Structures for CO Oxidation. *Sci. Rep.* **6**, 23900; doi: 10.1038/srep23900 (2016).

## Supplementary Material

Supplementary Information

## References

[b1] Antonaroli S. *et al.* Palladium complexes based nanogravimetric sensors for carbon monoxide detection. Sens. Actuator B-Chem. 208, 334–338 (2015).

[b2] McKinney P. V. Reduction of palladium oxide by carbon monoxide. J. Am. Chem. Soc. 54, 4498–4504 (1932).

[b3] Watanabe M., Uchida H., Igarashi H. & Suzuki M. Pt catalyst supported on zeolite for selective oxidation of CO in reformed gases. Chem. Lett. 21–22 (1995).

[b4] Igarashi H., Uchida H., Suzuki M., Sasaki Y. & Watanabe M. Removal of carbon monoxide from hydrogen-rich fuels by selective oxidation over platinum catalyst supported on zeolite. Appl. Catal. A-Gen. 159, 159–169 (1997).

[b5] Li G., Li L., Jiang D., Lia Y. & Shi J. One-pot synthesis of *meso*-structured Pd-CeO_x_ catalyst for efficient low-temperature CO oxidation under ambient conditions. Nanoscale 7, 5691–5698 (2015). 25744277 10.1039/c4nr07257j

[b6] López J. M. *et al.* Au deposited on CeO_2_ prepared by a nanocasting route: A high activity catalyst for CO oxidation. J. Catal. 317, 16–175 (2014).

[b7] Sudarsanam P. *et al.* Nano-Au/CeO_2_ catalysts for CO oxidation: Influence of dopants (Fe, La and Zr) on the physicochemical properties and catalytic activity. Appl. Catal. B-Environ. 144, 900–908 (2014).

[b8] Comotti M., Li W.-C., Spliethoff B. & Schüth F. Support effect in high activity gold catalysts for CO oxidation. J. Am. Chem. Soc. 128, 917–924 (2006). 16417382 10.1021/ja0561441

[b9] Ševčíková K. *et al.* Impact of Rh-CeO_x_ interaction on CO oxidation mechanisms. Appl. Surf. Sci. 332, 747–755 (2015).

[b10] Rodriguez J. A. *et al.* Active gold-ceria and gold-ceria/titania catalysts for CO oxidation: From single-crystal model catalysts to powder catalysts. Catal. Today 240, 229–235 (2015).

[b11] Qi J. *et al.* Facile synthesis of core-shell Au@ CeO_2_ nanocomposites with remarkably enhanced catalytic activity for CO oxidation. Energ. Environ. Sci. 5, 8937–8941 (2012).

[b12] Xie Q. S. *et al.* Facile preparation of well-Dispersed CeO_2_-ZnO composite hollow microspheres with enhanced catalytic activity for CO oxidation. ACS Appl. Mater. Inter. 6, 421–428 (2014). 10.1021/am404487b 24303982

[b13] Chen S. X., Zhao S. F., Xu Z., Liu Z. G. & Zhu R. L. Influence of pH on the catalytic performance of CuO-CoO_x_-CeO_2_ for CO oxidation. RSC Adv. 5, 61735–61741 (2015).

[b14] Hinokuma S., Yamashita N., Katsuhara Y., Kogami H. & Machida M. CO oxidation activity of thermally stable Fe-Cu/CeO_2_ catalysts prepared by dual-mode arc-plasma process. Catal. Sci. Technol. 5, 3945–3952 (2015).

[b15] Xie X., Li Y., Liu Z.-Q., Haruta M. & Shen W. Low-temperature oxidation of CO catalysed by Co_3_O_4_ nanorods. Nature 458, 746–749 (2009). 19360084 10.1038/nature07877

[b16] Li Y., Peng H., Xu X., Peng Y. & Wang X. Facile preparation of mesoporous Cu-Sn solid solutions as active catalysts for CO oxidation. RSC Adv. 5, 25755–25764 (2015).

[b17] Gonçalves R. V. *et al.* Easy access to metallic copper nanoparticles with high activity and stability for CO oxidation. ACS Appl. Mater. Interfaces 7, 7987–7994 (2015). 25816196 10.1021/acsami.5b00129

[b18] Gardner S. D. *et al.* Catalytic behavior of noble metal/reducible oxide materials for low-temperature carbon monoxide oxidation. 2. Surface characterization of gold/manganese oxide. Langmuir 7, 2140–2145 (1991).

[b19] Shinde V. M. & Madras G. Kinetics of carbon monoxide oxidation with Sn_0.95_M_0.05_O_2-delta_ (M = Cu, Fe, Mn, Co) catalysts. Catal. Sci. Technol. 2, 437–446 (2012).

[b20] Venkataswamy P., Jampaiah D., Lin F., Alxneit I. & Reddy B. M. Structural properties of alumina supported Ce-Mn solid solutions and their markedly enhanced catalytic activity for CO oxidation. Appl. Surf. Sci. 349, 299–309 (2015).

[b21] Jia C.-J. *et al.* Co_3_O_4_-SiO_2_ nanocomposite: a very active catalyst for CO oxidation with unusual catalytic behavior. J. Am. Chem. Soc. 133, 11279–11288 (2011). 21696181 10.1021/ja2028926

[b22] Hardacre C., Ormerod R. M. & Lambert R. M. Platinum-promoted catalysis by ceria: a study of carbon monoxide oxidation over Pt (111)/CeO_2_. J. Phys. Chem. 98, 10901–10905 (1994).

[b23] Liu W. & Flytzani-Stephanopoulos M. Total oxidation of carbon-monoxide and methane over transition metal-fluorite oxide composite catalysts. 2. Catalysts characterization and reaction-kinetics. J. Catal. 153, 317–332 (1995).

[b24] Fu Q., Saltsburg H. & Flytzani-Stephanopoulos M. Active nonmetallic Au and Pt species on ceria-based water-gas shift catalysts. Science 301, 935–938 (2003). 12843399 10.1126/science.1085721

[b25] Jasinski P., Suzuki T. & Anderson H. U. Nanocrystalline undoped ceria oxygen sensor. Sens. Actuator B-Chem. 95, 73–77 (2003).

[b26] Madier Y., Descorme C., Le Govic A. M. & Duprez D. Oxygen mobility in CeO_2_ and CexZr_(1−x)_O_2_ compounds: Study by CO transient oxidation and ^18^O/^16^O isotopic exchange. J. Phy. Chem. B 103, 10999–11006 (1999).

[b27] Esch F. *et al.* Electron localization determines defect formation on ceria substrates. Science 309, 752–755 (2005). 16051791 10.1126/science.1111568

[b28] Mogensen M., Sammes N. M. & Tompsett G. A. Physical, chemical and electrochemical properties of pure and doped ceria. Solid State Ionics 129, 63–94 (2000).

[b29] Collins S. *et al.* Effect of gallia doping on the acid-base and redox properties of ceria. Appl. Catal. A-Gen. 388, 202–210 (2010).

[b30] Holmgren A., Andersson B. & Duprez D. Interactions of CO with Pt/ceria catalysts. Appl. Catal. B-Environ. 22, 215–230 (1999).

[b31] Higashi K., Sonoda K., Ono H., Sameshima S. & Hirata Y. Synthesis and sintering of rare-earth-doped ceria powder by the oxalate coprecipitation method. J. Mater. Res. 14, 957–967 (1999).

[b32] Kang S. H., Sung Y.-E. & Smyrl W. H. The effectiveness of sputtered PtCo catalysts on TiO_2_ nanotube arrays for the oxygen reduction reaction. J. Electrochem. Soc. 155, B1128–B1135 (2008).

[b33] Rioux R. *et al.* Monodisperse platinum nanoparticles of well-defined shape: synthesis, characterization, catalytic properties and future prospects. Top. Catal. 39, 167–174 (2006).

[b34] Heinrichs B. T., Delhez P., Schoebrechts J.-P. & Pirard J.-P. Palladium-silver sol-gel catalysts for selective hydrodechlorination of 1, 2-dichloroethane into ethylene. J. Catal. 172, 322–335 (1997).

[b35] Wang J. A. *et al.* New insights into the defective structure and catalytic activity of Pd/ceria. Chem. Mater. 14, 4676–4683 (2002).

[b36] Zhou L. *et al.* Monodisperse, nanoporous ceria microspheres embedded with Pt nanoparticles: general facile synthesis and catalytic application. Rsc Adv. 4, 42965–42970 (2014).

[b37] He J. *et al.* Fabrication of monodisperse porous zirconia microspheres and their phosphorylation for Friedel-Crafts Alkylation of indoles. ACS Appl. Mater. Inter. 6, 2718–2725 (2014). 10.1021/am405202d 24447149

[b38] Sudarsanam P. *et al.* Highly efficient cerium dioxide nanocube-based catalysts for low temperature diesel soot oxidation: the cooperative effect of cerium- and cobalt-oxides. Catal. Sci. Technol. 5, 3496–3500 (2015).

[b39] Elias J. S., Risch M., Giordano L., Mansour A. N. & Yang S. H. Structure, bonding, and catalytic activity of monodisperse, transition-metal-substituted CeO_2_ nanoparticles. J. Am. Chem. Soc. 136, 17193–17200 (2014). 25406101 10.1021/ja509214d

[b40] Liu Y.-M. *et al.* Highly selective Ce-Ni-O catalysts for efficient low temperature oxidative dehydrogenation of propane. Catal Lett. 130, 350–354 (2009).

[b41] Reddy B. M. & Rao K. N. Copper promoted ceria-zirconia based bimetallic catalysts for low temperature soot oxidation. Catal. Commun. 10, 1350–1353 (2009).

[b42] Sayle T. X. T., Parker S. C. & Catlow C. R. A. The role of oxygen vacancies on ceria surfaces in the oxidation of carbon monoxide. Surf. Sci. 316, 329–336 (1994).

[b43] Jeong D.-W. *et al.* Comparative study on cubic and tetragonal Cu-CeO_2_-ZrO_2_ catalysts for water gas shift reaction. J. Ind. Eng. Chem. 27, 35–39 (2015).

[b44] Wu G., Guan N. & Li L. Low temperature CO oxidation on Cu-Cu_2_O/TiO_2_ catalyst prepared by photodeposition. Catal. Sci. Technol. 1, 601–608 (2011).

[b45] Kang M., Song M. W. & Lee C. H. Catalytic carbon monoxide oxidation over CoO_x_/CeO_2_ composite catalysts. Appl. Catal. A-Gen. 251, 143–156 (2003).

