# Supplementary material for: Transition-Metal Doped Ceria Microspheres with Nanoporous Structures for CO Oxidation
Source: Sci Rep. 2016 Mar 31;6:23900. doi: 10.1038/srep23900 (PMC4814921; doi:10.1038/srep23900)
Supplement: Supplementary Information [file srep23900-s1.pdf]

Support information

Transition-Metal Doped Ceria Microspheres with Nanoporous Structures for CO Oxidation

Lin Zhou [1], Xiaoxiao Li [2], Ze Yao [4], Zhuwen Chen [1], Mei Hong [1], Rongshu Zhu [4], Yongye Liang [2], Jing Zhao [1, 3]

[1] Guangdong Key Lab of Nano-Micro Materials Research, School of Chemical Biology &Biotechnology, Peking University Shenzhen Graduate School, Shenzhen, 518055, China

[2] Department of Materials Science & Engineering, South Unversity of Science & Technology of China, Shenzhen, 518055, China.

[3] State Key Laboratory of Pharmaceutical Biotechnology, Institute ofChemistry and Biomedical Sciences, School of Life Sciences, NanjingUniversity, Nanjing, 210093, China.

[4] Environmental Science and Engineering Research Center, School of Civil and Environmental Engineering, Harbin Institute of Technology Shenzhen Graduate School, Shenzhen, 518055, China

General Procedures

Sulfonation of porous polymer microspheres

To obtain sulfonated polymer microspheres, 2.0 g of Poly (GMA-*co*-EGDMA) microspheres was dispersed in 50 mL deionized water, followed by the addition of 9 g of Na_2SO_3 in 10 mL deionized water. The resulting transparent solution was stirred in oil bath at 70 ^oC for 24 hours. The white microspheres with sulfonated groups on the surface were obtained by filtration and then washed repeatedly with deionized water and ethanol. After that, the microspheres were dried in the oven at 60 ^oC overnight, which were denoted as sulfonated polymer microspheres.

Preparation of Monodisperse Porous Ceria Microspheres

To a suspension of 1g of the sulfonated polymer microspheres in 5 mL of water, 2 g of $Ce(NO_3)_3 \cdot 6H_2O$ were added. The mixture was transferred to oven set and then heated at 60 ^oC for 6 h. Then, the poly(GMA-*co*-EGDMA)/cerium microspheres were calcined at 600 ^oC for 12 h. Finally, monodisperse porous ceria microspheres were obtained.

Preparation of Monodisperse Porous Ceria Microspheres Substituted with Cu

To a suspension of 1g ofthe sulfonated microspheres in 5 mL water, 2 g of $Ce(NO_3)_3 \cdot 6H_2O$ was added into the mixture. At the same time, 64.3 mg of $CuSO_4 \cdot 5H_2O$ were added.The mixed solution was transferred to oven set at 60 ^oC and then heat-treated for 6 h. Finally, the obtained poly (GMA-*co*-EGDMA)/cerium microspheres with Cu^{2+} were calcined at 600 ^oC for 12 h. After that,

Cu_xCeO_{2-X}microspheres were obtained. The microspheres were then further activated at 330 ^oC for 3 h in flowing H_2/N_2 (0.5/99.5 v/v) at a heating rate of 10 ^oC/min.

Preparation of Monodisperse Porous Ceria Microspheres Substituted with Co

To a suspension of 1g of the sulfonated microspheres in 5 mL water, 2 g of $Ce(NO_3)_3$ 6H_2O and 68.0 mg of $Co(OAc)_2{\cdot}4H_2O$ were added. The mixed suspension was transferred to oven and then heat-treated at 60 ^oC for 6 h. The obtained polymer/cerium microspheres susbstituted with Co^{2+} were calcined at 600 ^oC for 12 h. After that, the Co_xCeO_{2-X} microspheres were treated at 330 ^oC for 3 h in flowing H_2/N_2 (0.5/99.5 v/v) at a heating rate of 10 ^oC /min.

Preparation of Monodisperse Porous Ceria Microspheres Substituted with Ni

1g of the sulfonated microspheres in 5 mL water was mixed with 2 g of $Ce(NO_3)_3$ 6H_2O, followed by the addition of 65.6 mg of $Ni(OAc)_2{\cdot}4H_2O$. The mixture was heat treated in the oven at 60 ^oC for 6 h. After calcination at 600 ^oC for 12 h, Ni_xCeO_{2-X} microspheres were obtained. Further activation was conductedat 330 ^oC for 3 h under the same reaction condition as Cu_xCeO_{2-X}.

Preparation of Monodisperse Porous Ceria Microspheres Substituted with Fe

In the sol-gel process, 1g of the sulfonated microspheres, 2 g of $Ce(NO_3)_3$ 6H_2O and 103.3 mg of $Fe(NO_3)_3{\cdot}9H_2O$ were dispersed in 5 mL of H_2O. After thermally treatedd at 60 ^oC for 6 h, polymer/cerium/Fe^{3+} hybrids were obtained. The microspheres werethen calcined at 600 ^oC for 12 h. After heat-treated at 330 ^oC for 3 h in flowing H_2/N_2 (0.5/99.5 v/v), Fe_xCeO_{2-X} microspheres were synthesized.

Preparation of Monodisperse Porous Ceria Microspheres Substituted with Mn

1g of the sulfonated microspheres and 2 g of $Ce(NO_3)_3 \cdot 6H_2O$ were dispersed in 5 mL water. Also, 59.8 mg of $MnCl_2 \cdot 4H_2O$ was mixed with the above suspension. The mixture was put in the oven set at 60 °C for 6 h. After calcination at 600 °C for 12 h, the as-obtained Mn oxide substituted CeO_2 microspheres were further treated at 330 °C for 3 h in flowing H_2/N_2 (0.5/99.5 v/v) at a heating rate of 10 °C /min.

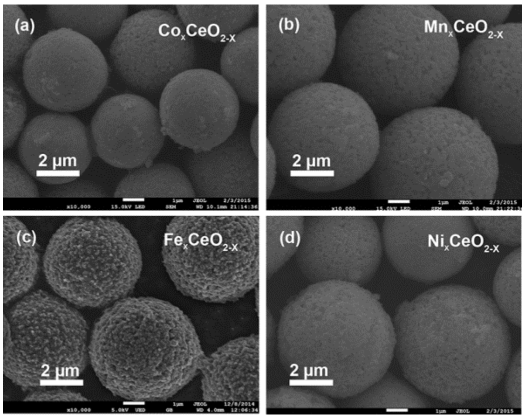

Supplementary Figure S1. SEM images of M_xCeO_{2-X} microspheres: (a) Co_xCeO_{2-X}, (b) Mn_xCeO_{2-X}, (c) Fe_xCeO_{2-X} and (d) Ni_xCeO_{2-X} microspheres.

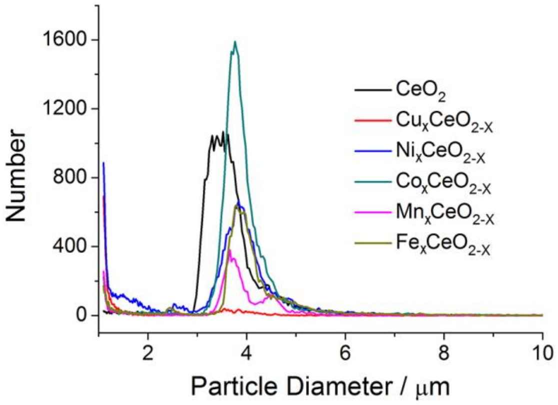

Supplementary Figure S2. The particle size distribution of CeO_2 microspheres (black line), Cu_xCeO_{2-X} microspheres (red line), Ni_xCeO_{2-X} microspheres (blue line), Co_xCeO_{2-X} microspheres (green line), Mn_xCeO_{2-X} microspheres (pink line) and Fe_xCeO_{2-X} microspheres (yellow green line).

Supplementary Table S1. Textural properties of metal substituted microspheres.

Entry	Microspheres	BET Surface Area (m^2/g)	BET Average PoreSize (nm)	t-Plot Micropore Area (m^2/g)	BJH Pore Volume (cm^3/g)	Metal Content (mol%)by ICP-OES	Metal Content (mol %) by EDS-mapping
1	Polymer	114	35.23	25	0.80	-	-
2	CeO_2	12	21.94	8	0.07	-	-
3	Cu_xCeO_{2-X}	9	21.88	5	0.06	10	9
4	Fe_xCeO_{2-X}	19	22.69	9	0.13	10	25
5	Co_xCeO_{2-X}	11	36.88	10	0.11	10	11
6	Ni_xCeO_{2-X}	17	24.01	8	0.12	10	11
7	Mn_xCeO_{2-X}	-		-	-	10	13

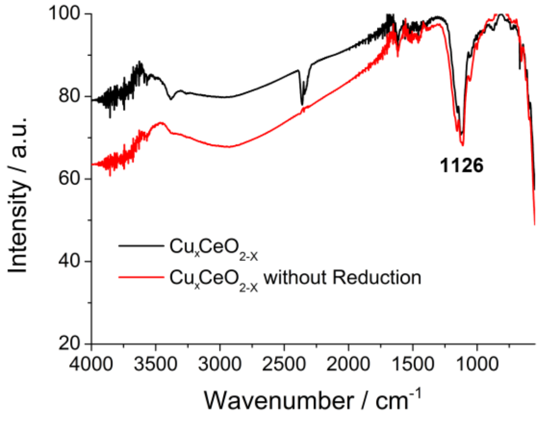

Supplementary Figure S3.DFTIR spectra of Cu_xCeO_{2-X} microspheres before and after H_2 reduction.

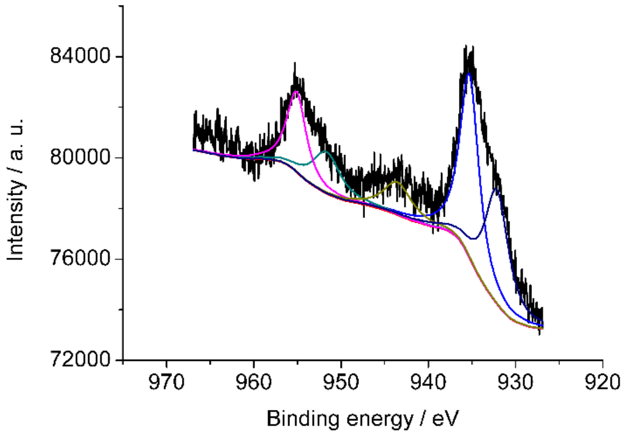

Supplementary Figure S4. XPS of Cu 2*p* region in Cu_xCeO_{2-X} microspheres.

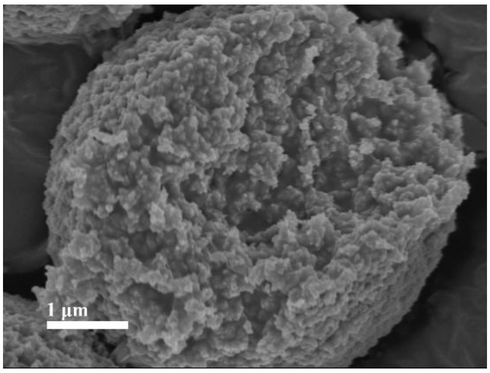

Supplementary Figure S5. SEM of an individual Cu_xCeO_{2-X} microspheres.

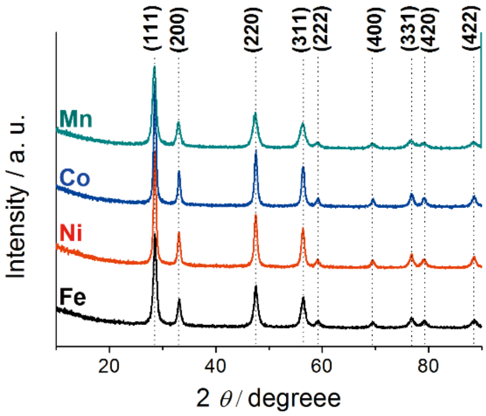

Supplementary Figure S6. XRD patterns of M_xCeO_{2-X} microspheres: Fe_xCeO_{2-X}, Ni_xCeO_{2-X}, Co_xCeO_{2-X} and Mn_xCeO_{2-X} microspheres.

Supplementary Table S2. Oxygen vacancy contents determined by XPS analysis of Ce 3*d* spectra.

Entry	Microspheres	Oxygen vacancy Content (%, XPS)
1	Cu_xCeO_{2-X}	14.5
2	Co_xCeO_{2-X}	11.4
3	Ni_xCeO_{2-X}	12.7
4	Mn_xCeO_{2-X}	13.6
5	Fe_xCeO_{2-X}	12.0

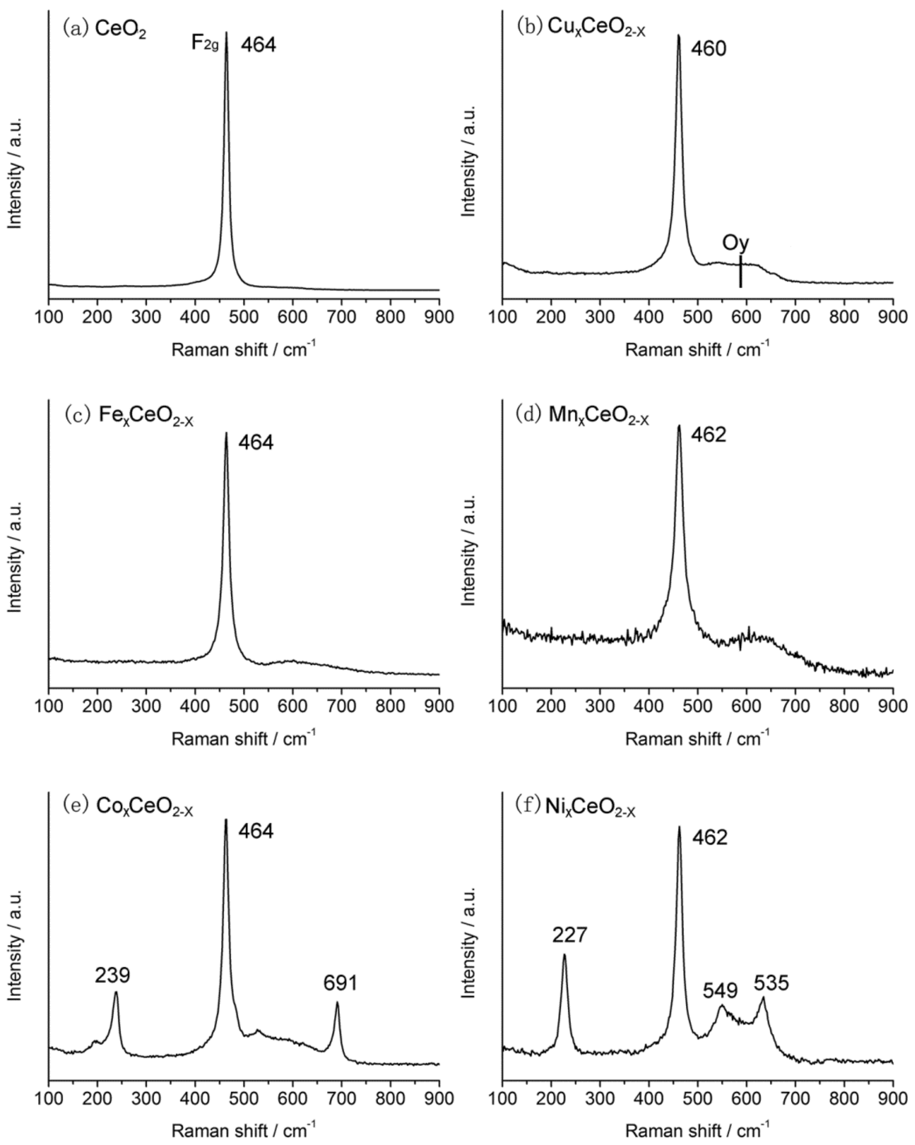

Supplementary Figure S7. Raman spectra of the metal substituted materials: (a) CeO_2, (b) Cu_xCeO_{2-X}, (c) Fe_xCeO_{2-X}, (d) Mn_xCeO_{2-X}, (e) $Co_xCeO_{2-X}$and (f) Ni_xCeO_{2-X}microspheres.

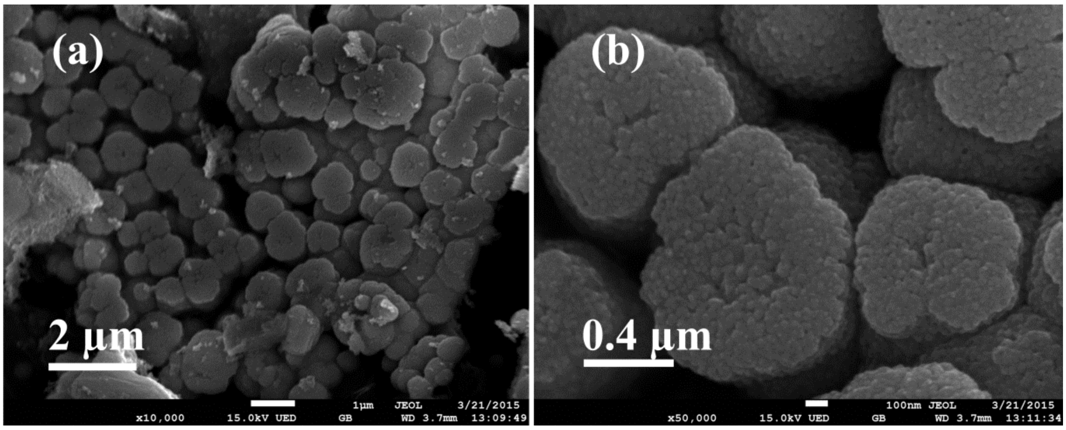

Supplementary Figure S8.SEM images of Cu_xCeO_{2-X} composite.

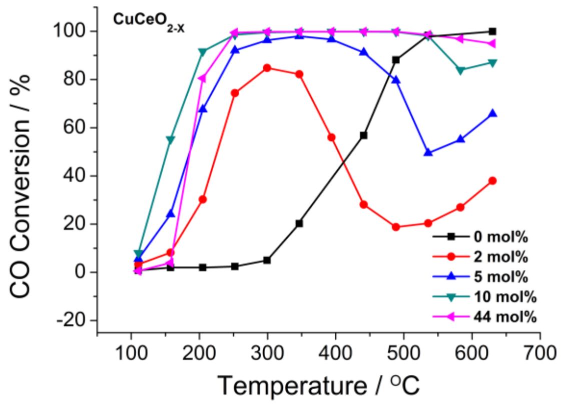

Supplementary Figure S9. CO catalytic oxidation on Cu_xCeO_{2-X} microspheres with different Cu contents.

Supplementary Table S3. Catalytic activities of different hybrid CeO_2 microspheres.

Entry	Microspheres	Special Treatment	Metal Content (mol%) by ICP-OES	T_{100} (°C)	T_{50} (°C)
1[a]	Cu_xCeO_{2-X}	Without Cu doping	0	520	421
2	Cu_xCeO_{2-X}	—	2	Above 600	226
3	Cu_xCeO_{2-X}	—	5	350	185
4	Cu_xCeO_{2-X}	—	10	300	150
5[b]	Cu_xCeO_{2-X}	Without template	10	300	218
6	Cu_xCeO_{2-X}	Without H_2 activation	10	300	231
7	Cu_xCeO_{2-X}	—	44	253	186
8	Fe_xCeO_{2-X}	—	10	Above 600	361
9	Ni_xCeO_{2-X}	—	10	350	223
10	Co_xCeO_{2-X}	—	10	300	216
11	Mn_xCeO_{2-X}	—	10	600	259

[a] Cu_xCeO_{2-X}without Cu doping was CeO_2 microsphere.

[b] Cu_xCeO_{2-X}without template was Cu_xCeO_{2-X} composite.

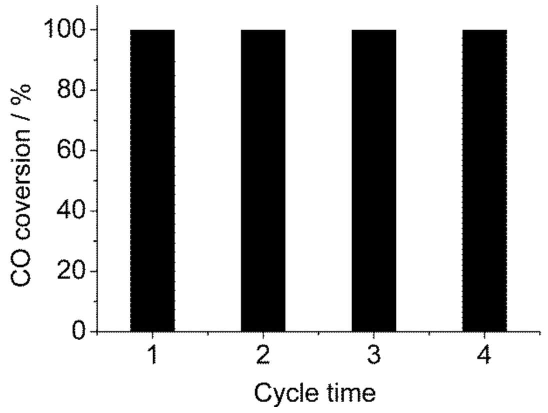

Supplementary Figure S10. Cycling CO conversion of Cu_xCeO_{2-X} microspheres.
